# Nutritional Intake and Biomarker Status in Strict Raw Food Eaters

**DOI:** 10.3390/nu14091725

**Published:** 2022-04-21

**Authors:** Klaus Abraham, Iris Trefflich, Fabian Gauch, Cornelia Weikert

**Affiliations:** Department of Food Safety, German Federal Institute for Risk Assessment, 10589 Berlin, Germany; iris.trefflich@bfr.bund.de (I.T.); fabian.gauch@web.de (F.G.); cornelia.weikert@bfr.bund.de (C.W.)

**Keywords:** strict raw food diet, vegans, omnivores, 3-day weighed food record, energy supply, biomarker, vitamin B12, homocysteine

## Abstract

Following a strict raw food diet (primarily based on fresh fruit and raw vegetables, waiving any consumption of heated or processed food) has the risk of undersupply of energy and certain macro- and micronutrients. In this cross-sectional study, we compared 16 non-smoking strict raw food eaters (5 women and 11 men, age 44.6 ± 12.3 years, duration of following the diet 11.6 ± 10.8 years) with the non-smoking participants (32 vegans, 27 omnivores) of the “Risk and Benefits of a Vegan Diet” (RBVD) study. We investigated body composition, dietary intake from 3-day weighed food records, and relevant fasting blood and serum parameters. Food choice and dietary behavior were very heterogenic in raw food eaters. They had lower mean values of BMI and percentage of body fat than the respective RBVD participants. The same holds true for energy supply and intakes of protein, carbohydrate, calcium and iodine. Serum levels revealed lower levels of HDL cholesterol, triglycerides, zinc, and vitamin D3. The raw food eaters with (*n* = 9) and without (*n* = 7) supplementation of vitamin B12 had median vitamin B12 levels of 399 and 152 ng/L, respectively. Accordingly, eight raw food eaters (50%) had homocysteine levels above 12 µmol/L. The study allows a close look at strict raw food eaters with respect to possible dietary deficiencies, but also provides insights into motivations and daily life.

## 1. Introduction

In order to modify and preserve the organoleptic and nutritional properties of foodstuffs, thermal processing has been carried out since humankind has learned to master fire for cooking purposes approximately 700,000 years ago. The benefits of this processing especially include inactivation of food-borne pathogens, destruction of natural toxins, prolongation of shelf life, improved digestibility and bioavailability of nutrients, improved palatability, taste, texture and flavour, as well as enhanced functional properties [1]. It is widely accepted that the continuous development of thermal food treatment had a substantial impact on phenotypical, intellectual, societal and economic development of humankind [2,3].

However, thermal processing can result in undesired consequences, such as loss of certain nutrients, or of the generation of compounds with negative effects on flavour perception, texture or colour [1]. Furthermore, toxic compounds may be formed during heating depending on the process temperature, especially through the Maillard reaction [4,5]. These heat-induced contaminants have gained high attention in research, risk assessment and risk management, especially acrylamide [6]. Other examples for these compounds are fatty acid esters of 3-MCPD and glycidol [7] or acrolein [8].

To strictly avoid the consumption of these undesired compounds, consumers would have to strictly refrain from heated food, eating raw food only, i.e., vegetables, fruits or grains as harvested, but also animal products like raw eggs, raw meat or raw fish would be possible to consume. Such a diet is expected to be very inconvenient for most people, and might be expected to result in nutritional undersupply, as, e.g., bread, potatoes or most dairy products have to be excluded from the diet. As a consequence, the intake of energy or vitamin B12 may be inadequate in the case of missing or only low consumption of raw animal products, as already reported for a study performed in Germany in the 1990s [9].

The primary aim of this study was to investigate the internal exposure to heat-induced compounds in strict raw food eaters, by quantifying specific mercapturic acids in urine as biomarkers of short-term exposure (e.g., [10]), and specific hemoglobin adducts as biomarkers of mid-term exposure (e.g., [11]). This aim is related to the question of sources possibly contributing to the internal exposure other than heated foods: endogenous metabolites formed by tissues or the gut microbiome, inhalational exposure and natural ingredients of foods are under discussion for several compounds [12,13]. The quantification of the corresponding biomarkers in strict raw food eaters is expected to provide informative answers helpful for the interpretation of biomarker levels of heat-induced compounds in populations with conventional dietary habits. These data will be published in separate articles.

In this article, we describe the nutritional intake and status of biomarkers of a group of 16 non-smoking raw food eaters strictly waiving any consumption of processed food and of food warmed/heated above 42 °C. They were compared to a group of 32 vegans and a group of 27 omnivores who did not avoid the consumption of heated or processed food. These groups consisted of the non-smoking participants of the “Risk and Benefits of a vegan diet” (RBVD) study [14].

## 2. Materials and Methods

### 2.1. Study Population

For this cross-sectional study, participants were mainly recruited at an exhibition for raw food nutrition (“Rohvolution”) in Berlin in March 2019 (www.rohvolution-messe.de, accessed on 23 March 2022). Inclusion criteria were healthy subjects (age between 20 and 65 years) following a strict raw food diet for at least four months, avoiding any consumption of food warmed up above 42 °C (home-prepared as well as industrially produced/processed food). Exclusion criteria were any smoking and any consumption of hot meals or beverages like coffee or tea. After an extensive interview regarding current and past dietary behavior, sixteen participants were included. They were invited to visit the study center at the German Federal Institute for Risk Assessment (BfR) in Berlin twice. At the first visit (between March and December 2019), they were provided with containers for 24 h urine collection and were instructed as to record their diet for three consecutive days. At the second visit, the participants gave one fasting blood sample, and nutritional and lifestyle characteristics were assessed with a questionnaire. Height, waist circumference, and blood pressure were measured using standardized methods. Body weight, fat and muscle mass were measured with a body composition analyzer (seca mBCA 515, seca GmbH, Hamburg, Germany). Physical activity was calculated according to the “recreational index” of the InterAct Consortium [15], which is based on sum duration of walking, cycling and other sports (averaged for summer and winter, in h/week) multiplied with standard metabolic equivalent of task (MET) estimates (3.0 for walking and 6.0 for cycling and other sports); it was categorized into inactive (≤33.75 MET-h/week) and active (>33.75 MET-h/week). The study was approved by the ethics committee of the Charité—Universitätsmedizin Berlin (No. EA4/040/19) and performed in accordance with the ethical standards laid down in the 1964 Declaration of Helsinki and its later amendments; it was registered in the German Clinical Trials Register (DRKS, No. 00017436).

The 16 strict raw food eaters were compared to non-smoking vegans (*n* = 32) and omnivores (*n* = 27) not avoiding the heating of food. These “control” subjects were participants of the “Risk and Benefits of a vegan diet” (RBVD) study also performed at the BfR (in 2017), and had to follow their diet for at least one year. In the RBVD study, an omni-vorous diet was defined as at least three servings of meat or two servings of meat and two servings of sausages a week. Detailed information about the RBVD study was published elsewhere [14].

### 2.2. Blood Sample and 24 h Urine Sample

The participants gave one fasting blood sample of 18 mL. Parameters of differential blood count and relevant serum parameters (e.g., glucose, lipids, vitamins, homocysteine) were analyzed on the same day at an accredited medical analytics laboratory (Labor 28 GmbH, Berlin, Germany). The remaining serum was fractionized and stored at −80 °C until further analysis. Urine was collected by the participants for 24 h, starting the day before the second study visit and ending with the first urine in the morning of the second visit.

### 2.3. Dietary Assessment

The participants were provided with digital kitchen scales and recorded their diet including beverages for three days before the second study visit (*n* = 15, one record was not provided). These 3-day weighed food records were used to gather detailed information on selected food items, portion sizes and meal pattern. Recorded data were transferred into the EAT software, version 3.5.5 (University of Paderborn, Paderborn, Germany), and each food item was assigned to the German national food code (Bundeslebensmittelschlüssel Version 3.02, BLS) to gain information about macro- and micronutrient intakes. Food items were categorized into food groups already existing in the RBVD study. Due to the characteristics of a raw food diet, germinated as well as fermented food, seaweed/algae and protein supplements were added as new food groups, thus 57 food groups were available in total. The dietary assessment was done according to methods of the RBVD study published elsewhere [16].

### 2.4. Statistics

Characteristics of the study population, laboratory parameters and intake of nutrients of strict raw food eaters and participants of the RBVD study were presented as means and standard deviation for normally distributed continuous variables or as median and interquartile range for non-normally distributed variables. Categorical variables are presented as percentages. For test of significance, Kruskal–Wallis or *t*-test for continuous variables were used. Differences between dietary intake and biomarkers in the three groups of diet were assessed using ANOVA and post hoc analysis. All analyses were stratified by sex, with a level of significance set at *p* < 0.05. All statistical analyses were conducted with SAS Enterprise software package version 9.3 (SAS Institute Inc., Cary, NC, USA). Serum levels below the limit of quantification (vitamin B12: *n* = 3; CrP: *n* = 11) were replaced by half the limit of quantification.

For the present study, no power calculations are provided. For the primary aim of this study (investigation of the internal exposure to heat-induced compounds), a number of at least 12 persons strictly consuming only unheated food was considered adequate, ideally with a balanced sex ratio.

## 3. Results

### 3.1. Study Population

Sixteen strict raw food eaters (5 women, 11 men) were included in the study. Individual characteristics like the duration of following a strict raw food diet are given in Table 1, together with a short description of the main food components usually eaten (interview/questionnaire data). In Table 2, basic data are presented separately for women and men; furthermore, the respective data for the (non-smoking) 32 vegans (18 women, 14 men) and 27 omnivores (14 women, 13 men) of the RBVD study are given. Compared to the latter groups, raw food eaters were a few years older, with an age of 44.6 ± 12.3 years (range 23 to 63 years). On average, they followed their diet for 11.6 ± 10.8 years (range 4 months to 29 years). In the five women, the duration of 3.9 ± 6.8 years (range 4 months to 16 years) was much shorter compared to that of the 11 men (15.1 ± 10.6 years, range 9 months to 29 years). Of the 16 participants, nine (56%, 8 men and 1 women) followed the strict raw food diet for 10 years or longer. Those who followed this diet for a short duration already followed a diet with at least about 80% raw food for at least several months beforehand.

Compared to the RBVD groups, female and male raw food eaters were found to have the lowest BMI of 21.4 ± 3.0 and 20.7 ± 2.0 kg/m^2^, respectively (Table 2). Three of the raw food eaters (19%, one women and two men) were underweight with a BMI below 18.5 kg/m^2^. In the RBVD study (*n* = 59), only one vegan male (1.7%) fell below this BMI cutoff. Accordingly, mean values of body fat (as percentage of body weight) was lowest in female and male raw food eaters, whereas the corresponding mean values of muscle (as percentage of body weight) were highest in female and male raw food eaters, but not that different to the respective other two groups each. Further sex-specific data of body parameters are given in Table 2. Regarding the physical activity defined as active (>33.75 MET-h/week), the lowest values of 20 and 70% were observed in female and male raw food eaters, respectively (Table 2). The proportion of a high educational attainment level (degree of university or university of applied sciences) was 60, 66 and 74% in raw food eaters, RBVD vegans and RBVD omnivores, respectively.

### 3.2. Dietary Intake

According to the information given by the participants, the main nutritional basis of nearly all 16 participants was the consumption of fresh fruit and raw vegetables; furthermore, eight participants (50%) stated nuts as an important food (Table 1). Four subjects (25%, 1 women, 3 men) followed a strict raw vegan diet. Four participants (25%, 2 women, 2 men) can be classified as vegetarian, occasionally also consuming raw milk cheese or raw eggs. Eight participants (50%, 2 women, 6 men) also ate raw meat and/or raw fish, at least occasionally, but in most cases not reaching the inclusion criteria for omnivores in the RBVD study (at least three servings of meat or two servings of meat and two servings of sausages a week). Twelve participants (75%) stated the intake of nutritional supplements. Vitamin B12 and D—taken at least occasionally or seasonally—were the mostly supplemented nutrients (*n* = 9 each, 56%). The corresponding proportions in RBVD vegans and omnivores were 94% and 11% for vitamin B12 as well as 50% and 11% for vitamin D, respectively. For individual data of the raw food eaters, see Table 1.

**Figure 1:**
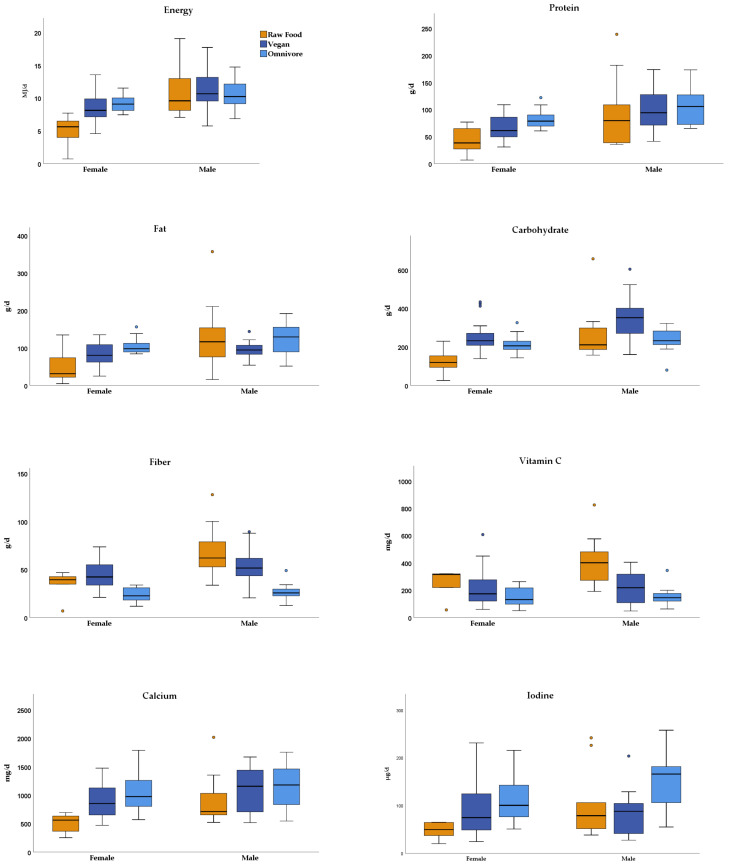
Intake of energy and selected nutrients in women and men of raw food eaters, RBVD vegans and RBVD omnivores. Median of energy (MJ/day), macronutrients (g/day) and micronutrients (mg/day or µg/day) are based on 3-day weighed food records. Coloured dots are outliers.

Data of the intake of nutrients based on 3-day weighed food records are presented in Table 1, Figure 1 and Supplementary Table S1 (*n* = 15, due to one missing record of a male raw food eater). With respect to the meal patterns, one striking observation was the unequal distribution of meals over the day in many participants, with missing breakfast and meals starting late in the day in 8 participants (53%). Furthermore, in part, extreme amounts of certain foods were eaten with single meals (see below). Median energy intake was found to be lowest in female and in male raw food eaters, as compared to the respective RBVD vegan and omnivore groups. A high daily fiber intake was observed especially in men (median 62 g/day compared to 52 and 26 g/day in RBVD vegans and omnivores, respectively). In raw food eating women and men, median daily protein intake was lower than that of RBVD vegans who, in turn, had a lower intake than RBVD omnivores. In women as well as in men, the median daily intake of carbohydrate was lowest in the raw food eaters and highest in the RBVD vegans. In women, the median daily intake of fat was distinctly lower in raw food eaters (median 31 g/day) compared to RBVD vegans and omnivores (median 80 and 98 g/day, respectively), while in men, the median fat intake of raw food eaters (116 g/day) was between those of RBVD vegans (95 g/day) and omnivores (129 g/day).

The intake data of selected food groups based on 3-day weighed food records are presented in Table 1 and Supplementary Table S2. Data demonstrate the relatively high intake of raw vegetables (median consumption of 310 g/day in women and 566 g/day in men, >1 kg/day in two participants), fresh fruits (median consumption of 409 g/day in women and 906 g/day in men, >1 kg/day in six participants), dried fruits (nearly 800 g/day in one participant), as well as nuts/seeds (>300 g/day in two participants). For the whole group of raw food eaters, median consumption of fresh fruit, raw vegetables and nuts/seeds was 3.6-, 3.3- and 3.1-fold higher as compared to the RBVD vegans, respectively. Food groups such as fermented vegetable, germinated cereals, or seaweed were only consumed by the raw food eaters. As expected, on the other hand, many food groups (34 of the 57 categories) were not consumed by anyone among the raw food eaters, e.g., pasta, bread, (cooked) legumes, tea or coffee. In order to give an impression of the amounts of these food groups consumed by the RBVD vegans and omnivores, the food groups “cooked vegetables”, “cooked legumes”, “bread” and “pasta/rice/grain” were additionally compiled in Supplementary Table S2, as examples for quantitatively relevant food groups not consumed by the raw food eaters. During the three record days, animal-based raw food was consumed by six (male) participants: three participants had eaten raw meat (3-day averages 35 g/day, 100 g/day, and in one case 1007 g/day horse meat), one participant had consumed raw eggs (53 g/day), raw meat (57 g/day) and raw milk (1140 mL/day), one participant had eaten mussel (56 g/day) and dried poultry (5 g/day), and one participant had consumed raw fish (306 g/day, by the way, 918 g with a single meal). The corresponding maximum consumption data of male RBVD omnivores for milk, meat and fish consisted of 505 mL/day, 200 g/day and 114 g/day, respectively.

With respect to micronutrients estimated from the 3-day weighed food records (Supplementary Table S1, selected parameters in Figure 1), the most striking observation was the high intake of vitamin C in the raw food eaters (median 315 mg/day in women and 401 mg/day in men), as compared to the RBVD groups with the highest median of 218 mg/day in vegan men. Regarding lower intake levels in the raw food eaters, the medium intake of calcium was lowest in the raw food women (561 mg/day) and men (710 mg/day), as compared to the RBVD groups with the lowest median levels in female (852 mg/day) and male (1156 mg/day) vegans. Furthermore, the medium intake of iodine was lowest in the raw food women (49.6 µg/day) and men (78.9 µg/day), as compared to the RBVD groups with the lowest median levels in female (74.8 µg/day) and male (88.0 µg/day) vegans. Due to the heterogeneity of food consumption in the raw food group, the intake of no other micronutrient was found to be consistently higher or lower in both sexes, as compared to the corresponding RBVD groups and to reference values of the nutrition societies of Germany, Austria, and Switzerland [17], additionally compiled in Supplementary Table S1.

### 3.3. Laboratory Blood and Serum Parameters

Parameters measured in serum are compiled in Table 3 for women and men. In comparison to the respective RBVD groups, consistently higher mean levels in female and male raw food eaters were observed for serum protein. Consistently lower median levels in female and male raw food eaters were observed for HDL cholesterol, gamma GT, GPT, triglycerides, zinc, and vitamin D3. Furthermore, lowest median vitamin B12 levels as well as highest homocysteine level were observed in female and male raw food eaters, as compared to the corresponding RBVD groups. The raw food eaters with (*n* = 9) and without (*n* = 7) supplementation of vitamin B12 had median vitamin B12 levels of 399 and 152 ng/L, and median homocysteine levels of 10.3 and 15.9 µmol/L, respectively. Three of the raw food eaters without supplementation had vitamin B12 levels below the limit of detection of 100 ng/L (not observed in the RBVD study); they all had homocysteine levels above 20 µmol/L. Eight participants (50%) had homocysteine levels above 12 µmol/L, whereas this was the case in 2 of the 32 RBVD vegans (6.3%) and 2 of the 27 RBVD omnivores (7.4%) only.

## 4. Discussion

In this paper, we report on a very special type of diet strictly avoiding any warming or heating of food above 42 °C, i.e., a temperature not leading to denaturation of proteins. The 16 strict raw food eaters recruited were found to be well informed, e.g., about natural ingredients of foods or about processes in food production (e.g., which foods have been heated before offered for sale). They were very convinced of this type of diet, which is apparent by the, in part, very long duration in which it is carried out, or the daily burden taken on: to obtain the right foods (e.g., growing of sprouts, shopping in specialized markets or via internet), or to refrain from many foods and the visits of most restaurants.

While the renunciation of heating of a single food in most cases has no direct consequences for the nutrition of an individual (not taking into account the inactivation of food-borne pathogens and the destruction of natural toxins), the strict renunciation of any warming/heating and processing has strong consequences, making a well-balanced nutrition with a great choice of food products difficult. This is mainly due to the fact that some harmful ingredients are only rendered harmless by cooking (e.g., potatoes), and that some foods get eatable after cooking or baking only (e.g., pasta, rice, bread, cakes, and likewise hot beverages, such as tea or coffee). As documented in the 3-day weighed food records, only 23 of 57 food groups have been consumed by at least one of the participants. On the other hand, single food groups have been eaten in large amounts, exceeding 1 kg per day in 9 participants (fresh fruit, raw vegetables, raw milk, or meat). Regarding qualitative aspects, a few raw food eaters consumed food commonly not eaten in large amounts, like certain exotic fruits (e.g., cherimoya, kumquat), seaweed or germinated vegetables. A high consumption (especially fresh fruit, raw vegetable and in part of nuts/seeds) was necessary to meet the individual requirements of energy supply, but nevertheless—in view of the BMI data—obviously was inadequate in the long run in a part of the raw food eaters. In line with the lower supply of energy, protein and carbohydrate intakes were lower in the raw food groups, whereas fat intake was lower in the female raw food eaters only.

Most participants were aware of the general risk of an insufficient supply of specific nutrients, but overwhelmingly felt to be adequately supplied with their individual diet, without having had an individual nutritional counseling or a blood test. With respect to the supply of vitamin B12 as the most critical nutrient in plant-based diets [14,18,19], only 56% of the raw food eaters took a vitamin B12 supplement (in some cases from time to time only), which is much lower in comparison to the RBVD vegans with a rate of 94%. Consequently, the lowest vitamin B12 serum levels and highest homocysteine levels were observed in female as well as in male raw food eaters, as compared to the respective RBVD groups. Three of the raw food eaters (19%, all without supplementation) had vitamin B12 levels below the limit of detection; eight (50%) had elevated homocysteine above 12 µmol/L, indicating a deficiency in vitamin B12, B6 and/or folic acid, and possibly an increased risk for cardiovascular diseases and diseases of the central nervous system [20]. Of the other laboratory serum parameters, the group differences observed in a part were not that relevant to draw conclusions related to health risks. Besides the relatively low number of participants, especially in the case of the raw food women, a comparison to the respective groups of the RBVD study should be made with caution. In particular, the inhomogeneity of the raw food eaters has to be considered with respect to the individually different duration of this diet, and with respect to the quantitative and qualitative differences regarding selection of foods (including the proportion of food of animal origin) and supplementation.

With respect to the motivation to follow a raw food diet, the main reasons reported by the participants were recommendations by friends, physicians or books, as well as positive perceptions and effects on different health conditions after starting the diet which have led to a high grade of conviction. Regarding scientific rationales, different reasons were mentioned by the participants for following the strict raw food diet, as missing denaturation and/or destruction of relevant food ingredients, and missing exposure to food additives as well as substances derived from nonenzymatic glycation reactions during industrial processing and home cooking—especially products of the Maillard reaction like acrylamide [5] and “Advanced Glycation End products” (AGEs) [21,22]. Some of the participants referred to the unproven concepts of the “instinctotherapy” by Guy-Claude Burger defined in 1964, as described by an article of Jallut [23]. This diet consists exclusively of food in its natural form (including raw meat and fish; no heating, preserving or seasoning; dairy products are not allowed). According to this theory, humankind originally ate raw food, and instincts have not evolved since those prehistoric times, but have been artificially modified by cooked food and consumption of non-human milk. Furthermore, it is assumed that there was not enough time during evolution to adapt the human metabolism to the compounds produced in heating processes, leading to their incomplete elimination from the body. According to Burger, health problems including cancer could be solved by re-developing natural instincts using the sense of smell and taste when selecting, e.g., a fruit for a meal. No breakfast is eaten apart from drinking little mineral water, justified with the believe of a “nocturnal detoxification,” which has not been finished in case of hunger in the morning [23]; this indeed was practiced by about half of the participants (Table 1).

Recent studies investigating nutritional intake and status of biomarkers in raw food eaters are rare. The largest (cross-sectional) study in this context is the “Gießen Raw Food Study” performed in Germany in the 1990s, however, with weaker criteria of inclusion. Questionnaire data of 216 men and 297 women consuming long-term raw food diets with an average duration of 3.7 years revealed an average weight loss from the beginning of the dietary regimen of 12 kg in women and of 9.9 kg in men. Underweight (BMI < 18.5 kg/m^2^) was observed in 25.0% of female and 14.7% of male subjects and was inversely related to the amount of raw food consumed and the duration of the raw food diet. Partial to complete amenorrhea was reported by about 30% of the women under 45 years of age [24]. The final study group consisted of 201 non-smoking raw food eaters (107 women and 94 men) with median duration of the diet of 3.5 years and a mean age of 46 years comparable to our study [9]. They consumed at least 70% raw food, and 57 participants followed a strict raw food diet. As in our study, the diets were classifiable as vegan, ovo-lacto-vegetarian or mixed; no control group with conventional diets was established. A 7 d-estimated and self-administrated food record revealed median intakes in these three groups between 1029 and 1349 g/day in the case of fruits (compared to 635 g/day in our group), and between 411 and 457 g/day in the case of raw vegetable (compared to 482 g/day in our group). The median energy intake of the three groups was between 7.5 and 8.6 MJ/day, as compared to 5.6 and 9.6 MJ/day in our female and male raw food eaters, respectively (Supplementary Table S1). Regarding the laboratory serum parameters, the main findings of the “Gießen Raw Food Study” were relatively low levels of HDL cholesterol and vitamin B12, as well as relatively high levels of homocysteine, which were inversely correlated with the vitamin B12 levels. The same trends of these parameters were observed in our study.

A small cross-sectional study [25] investigated a group of 18 strict raw food vegetarians with a mean duration of the diet of 3.6 years and a mean age of 54 years (7 women, 11 men). They were compared to a control group matched for age, sex, and socioeconomic status, eating a typical American diet. The BMI was significantly lower in the raw food eaters (mean 20.5 kg/m^2^) as compared to the controls (mean 25.4 kg/m^2^). Bone mineral content (BMC) and bone mineral density (BMD) of the total body, lumbar spine, and proximal femur were measured by dual-energy X-ray absorptiometry, revealing significantly lower values for these parameters in the raw food eaters at all sites. In contrast, serum markers of bone turnover were not significantly different.

Regarding limitations, the strict raw food eaters of this study are a small group only, but the group consists of people completely avoiding any heating of food. In general, a group of raw food eaters is expected to be very heterogeneous with respect to the individual duration of the diet, to the proportion of food of animal origin consumed, to the extent of supplementation, and to quantitative and qualitative differences in selection of food. Therefore, a higher number of participants would be helpful to receive results, which are more meaningful, but as only few people follow this diet nowadays, a larger group would be challenging to recruit. A strength of the present study is the possibility of a direct comparison of raw food eaters to people also eating heated food following a vegan or an omnivorous diet. With the use of 3-day weighed food records, the diet has been assessed in detail, providing information on selected food items, portion sizes and meal pattern, allowing evaluation of the heterogeneity of food groups and estimation of energy and nutrient intake over time in comparison to the vegan and omnivore “controls”. Compared to other methods of dietary assessment such as food frequency questionnaire, a weighed food record is the most appropriate instrument to assess the diet in raw food eaters, especially as pre-specified food groups could hardly account for the specific characteristics of this diet. Together with the questionnaire and blood/serum data, the study allows a close look on strict raw food eaters with respect to possible dietary deficiencies, but also provides insights into motivations and their daily life.

## 5. Conclusions

Despite the low numbers of raw food participants, our investigations allow the consideration of consequences of a strict raw food diet waiving any warming/heating and any processing of food. As is already well known, an insufficient supply of vitamin B12 is expected to be the main harm in the case of a raw vegan diet without or insufficient supplementation, and in the case of a raw vegetarian or mixed diet with insufficient consumption of food of animal origin. This was quantifiable in homocysteine levels above 12 µmol/L observed in 50% of the participants. The deficiency could easily be avoided by vitamin B12 supplementation, as demonstrated by the RBVD vegans with a supplementation rate of 94% and a rate of elevated homocysteine levels of 6%.

A second important issue is the difficulty to ensure an adequate supply of energy in a raw food diet. This becomes evident by the low BMIs observed. To reach an adequate energy supply, the limited choices of food groups available require the consumption of higher amounts of certain food. The long-term consequences of this qualitatively and, in part, quantitatively reduced food supply have to be investigated in more detail. Currently, a higher proportion of underweight and amenorrhea in women [24] as well as a lower bone mass [25] have been observed. With respect to laboratory parameters investigated in our study, most levels were found to lay within the normal range, even in subjects with a duration of a raw food diet of 10 years and longer.

## Figures and Tables

**Table 1:** Individual characteristics of the 16 strict raw food eaters.

		Interview/Questionnaire		3-Day Weighed Food Record (*n* = 15)	Suppl.	
Age	Sex, Duration of Raw Food Diet (Years)	Type of Diet	Main Nutritional Components in Brackets: Food of Animal Origin	Striking Observations Meal Pattern	Vitamin B12	Vitamin D
**20–29 years**	male, 1.5	mixed	Fruits, vegetable, nuts, avocado (egg 1–2 ×/week, infrequent: meat, fish)	Germinated legumes/cereals, meat meals start late in the day		
	female, 2.3	vegetarian	Red millet, buckwheat, lentils, wild herbs, sprouts, fruits (raw milk cheese every 2 weeks)	Seaweed, germinated legumes/cereals	✓	✓
	male, 1.8	vegan	Fruits, vegetable, nuts	Fresh fruits >1 kg/day, protein bars, fermented vegetables	✓	✓*
**30–39 years**	male, 0.75	vegan	Vegetable, fruits, sprouts, wild herbs	Nuts >300 g/day, raw vegetables >1 kg/day meals start late in the day	✓	✓
	male, 10	vegetarian	Red millet, buckwheat, lentils, wild herbs, sprouts, fruits (raw milk cheese every 2 weeks)	Seaweed, germinated legumes/cereals	✓	✓
**40–49 years**	male, 28	mixed	Fruits, nuts, avocado, meat (meat, fish, egg 1–2 ×/week)	Fresh fruits >1 kg/day, germinated legumes/cereals, mussel, poultry meals late in evening and night		✓*
	male, 26	vegetarian	Fruits, vegetable, nuts (infrequent: egg)	No record delivered	✓	✓*
	female, 0.33	vegan	Vegetable, salads	Meals start late in the day, one fasting day	✓	✓*
	male, 14	mixed	Fruits, vegetables, nuts, meat (up to 1 kg/week) (meat, fish, infrequent: egg, liver)	Fresh fruits >1 kg/day, meat meals start late in the day		
	female, 16	mixed	Peas, protein bars, nuts (meat, fish, egg)	Protein bars	✓	
**>50 years**	male, 15	mixed	Products of raw milk, fruits, salads with vegetable (meat, liver, egg, milk)	Milk >1 L/day, meat, eggs meals start late in the day	✓	
	male, 29	mixed	Fruits, vegetable, nuts (meat, fish)	Fish >300 g/day, nuts >300 g/day, raw vegetables >1 kg/day meals start late in the day		
	female, 0.33	mixed	Fruits, vegetable (infrequent: fish)	Fresh fruits >1 kg/day	✓	✓
	male, 20	vegan	Nuts, vegetable, pumpkin, mushrooms, fruits	Fresh fruits >1 kg/day meals start late in the day		
	female, 0.33	vegetarian	Vegetable, fruits, buckwheat, raw milk butter	Fermented vegetables		✓
	male, 20	mixed	Fruits, salads, (meat)	Fresh fruits >1 kg/day, meat >1 kg/day		

* in winter only.

**Table 2:** Characteristics of study populations: women and men of raw food eaters, RBVD vegans and RBVD omnivores.

	Women			Men		
	Raw Food (*n* = 5)	Vegan (*n* = 18)	Omnivore (*n* = 14)	Raw Food (*n* = 11)	Vegan (*n* = 14)	Omnivore (*n* = 13)
Age (years)	44.8 ± 14	38.9 ± 7.8	38.6 ± 8.3	44.6 ± 12.2	37.6 ± 8.0	40.2 ± 7.2
BMI (kg/m^2^)	21.4 ± 3.0	21.8 ± 3.0	23.2 ± 2.2	20.7 ± 2.0	24.5 ± 3.3	24.4 ± 1.8
Waist (cm)	74.0 (72.0–74.0)	71.9 (68.2–75.8)	73.8 (72.8–76.0)	76.0 (74.0–86.0)	86.3 (79.9–92.4)	86.8 (83.7–90.5)
Weight (kg)	60.2 (55.8–65.0)	59.3 (52.4–67.8)	65.6 (61.3–72.4)	65.0 (60.7–71.3)	81.9 (72.8–92.7)	81.1 (78.0–85.9)
Duration Raw (years)	3.9 ± 6.8			15.1 ± 10.6		
Body fat (% of body weight)	28.7 ± 8.2	29.1 ± 6.5	30.8 ± 4.9	13.9 ± 4.9 ***	20.8 ± 4.6	20.8 ± 2.9
Muscle (% of body weight)	30.6 ± 2.9	29.6 ± 2.8	29.7 ± 2.6	39.8 ± 3.4 ***	37.4 ± 2.5	37.2 ± 2.2
Blood pressure Diast. (mm Hg)	71.4 ± 14.7	68.5 ± 7.7	70.8 ± 5.3	69.2 ± 8.5	71.9 ± 8.0	76.6 ± 7.7
Blood pressure Syst. (mm Hg)	110.0 ± 12.4	105.7 ± 8.4	106.1 ± 8.5	117.6 ± 13.6	117.6 ± 11.9	123.2 ± 11.8
High physical activity (*n*) *	1 (20%)	16 (89%)	12 (86%)	7 (70%) ****	12 (86%)	12 (92%)
High education (*n*) **	4 (80%)	12 (67%)	9 (64%)	5 (50%) ****	9 (64%)	11 (85%)
Any supplementation (*n*)	5 (100%)	17 (94%)	6 (43%)	7 (64%)	14 (100%)	4 (31%)

Continuous data presented as mean and standard deviation (±) or median and interquartile range (Q1–Q3). Categorical variables were presented as absolute numbers. * Physical activity is defined as active (>33.75 MET-h/week), based on categories of recreational index. ** High education was defined as university and university of applied science. *** *n* = 8. **** *n* = 10.

**Table 3:** Laboratory blood and serum parameters of women and men of raw food eaters, RBVD vegans and RBVD omnivores.

	Women			Men		
	Raw Food (*n* = 5)	Vegan (*n* = 18)	Omnivore (*n* = 14)	Raw Food (*n* = 11)	Vegan (*n* = 14)	Omnivore (*n* = 13)
Haemoglobin [g/dL]	13.5 ± 0.7	12.8 ± 0.8	12.7 ± 0.9	13.5 ± 1.5 ^a,c^	14.5 ± 0.9 ^a^	15.0 ± 0.6 ^a,c^
Haematocrit [%]	40.1 ± 2.7	39.0 ± 2.2	38.8 ± 2.7	40.8 ± 4.3	42.8 ± 2.6	43.6 ± 1.8
Erythrocytes [T/L]	4.6 ± 0.3	4.3 ± 0.5	4.4 ± 0.3	4.5 ± 0.5	4.9 ± 0.4	5.0 ± 0.3
MCV [fl]	88.1 (87.5–88.9) ^a,b^	91.4 (89.3–92.4) ^a,b^	90.0 (88.8–91.2) ^a^	90.1 (88.8–93.7)	88.2 (87.2–92.2)	88.8 (84.0–89.5)
MCHC [g/dL]	33.6 ± 0.9	32.8 ± 0.7	32.7 ± 1.1	33.1 ± 0.5	34.0 ± 0.6	34.4 ± 0.7
Leucocytes [G/L]	3.8 (3.8–5.9)	5.2 (3.7–6.8)	4.8 (4.5–5.7)	5.0 (4.2–6.1)	4.6 (4.1–5.6)	4.7 (4.5–5.7)
Thrombocytes [G/L]	225 (216–269)	225 (191–299)	241 (220–276)	247 (199–290)	196 (147–220)	217 (184–231)
Protein total [g/dL]	7.1 ± 0.5	6.9 ± 0.3	7.0 ± 0.4	7.5 ± 0.4 ^a,b,c^	6.8 ± 0.5 ^a,b^	7.0 ± 0.3 ^a,c^
CRP [mg/L]	0.30 (0.30–0.30)	0.29 (0.19–0.47)	0.47 (0.08–0.75)	0.30 (0.30–0.90)	0.79 (0.39–1.06)	0.63 (0.26–1.59)
Glucose [mg/dL]	74 (69–80)	82 (76–85)	79 (75–85)	82 (79–89)	81 (79–88)	84 (83–91)
HbA1c [%]	5.3 (5.1–5.3)	5.1 (5.0–5.2)	5.1 (5.1–5.2)	5.1 (5.0–5.3)	5.1 (5.0–5.2)	5.2 (5.1–5.4)
gamma GT [U/L]	11 (11–14)	12 (10–17)	13 (10–17)	13 (12–23) ^a,c^	16 (13–22) ^a^	24 (18–29) ^a,c^
GPT [U/L]	15 (12–22)	18 (15–21)	16 (13–18)	20 (18–24)	25 (19–35)	27 (21–37)
Creatinine [mg/dL]	0.71 ± 0.06	0.73 ± 0.09	0.83 ± 0.09	0.89 ± 0.19	0.93 ± 0.13	1.01 ± 0.14
TSH [mU/L]	1.91 (1.88–1.94)	1.86 (1.28–2.33)	2.18 (1.64–3.33)	1.42 (1.10–3.12)	2.30 (1.72–3.25)	2.42 (1.95–3.04)
Ferritin [ng/mL]	76 (53–97) ^a,b^	34 (17–42) ^a,b^	40 (22–70) ^a^	110 (45–168)	81 (67–86)	121 (68–153)
Cholesterol [mg/dL]	172 (154–185) ^a^	160 (139–177) ^a,d^	198 (186–218) ^a,d^	131 (122–163) ^a,c^	156 (133–212) ^a^	195 (168–215) ^a,c^
LDL [mg/dL]	94 (94–111)	91 (73–96)	104 (84–116)	70 (62–94) ^a,c^	85 (70–125) ^a^	116 (93–136) ^a,c^
HDL [mg/dL]	54 (51–71) ^a^	65 (56–74) ^a,d^	80 (70–83) ^a,d^	49 (46–52)	51 (45–56)	52 (48–60)
Triglycerides [mg/dL]	51 (38–68)	64 (51–74)	55 (47–93)	62 (51–101)	85 (61–111)	86 (70–136)
Vitamin B12 [pg/mL]	329 (280–399)	449 (312–657)	383 (317–521)	290 (152–433)	673 (288–792)	431 (361–488)
Homocysteine [µmol/L]	10.3 (6.7–15.5)	8.6 (6.6–10.2)	7.7 (6.9–8.6)	12.2 (8.9–19.0) ^a,b^	8.3 (7.8–11.3) ^a,b^	9.8 (8.8–10.8) ^a^
Vitamin D3 [mmol/L]	37.2 (28.2–43.9)	52.2 (23.9–84.2)	51.8 (28.4–81.8)	37.0 (26.7–56.6)	68.6 (18.6–108.5)	41.4 (36.3–59.2)
Calcium [mmol/L]	2.35 ± 0.09	2.33 ± 0.06	2.35 ± 0.07	2.40 ± 0.12	2.36 ± 0.09	2.39 ± 0.05
Zinc [µg/dL]	76 (69–81)	81 (72–89)	82 (79–91)	71 (64–88)	77 (75–90)	84 (80–103)

Data presented as means ± SD or median (Q1–Q3). Values sharing a common superscript letter were significantly different from each other. ANOVA with post hoc analysis (*p* < 0.05): ^a^ significant differences within sex strata; ^b–d^ significant differences between type of diet.

## Data Availability

The datasets generated during and/or analyzed during the raw food and the RBVD study are not publicly available due to provisions of the data protection regulations.

## Supplementary Materials

The following supporting information can be downloaded at: https://www.mdpi.com/article/10.3390/nu14091725/s1, Table S1: Nutrient intake based on 3-day weighed food records in women and men of raw food eaters, RBVD vegans and RBVD omnivores; Table S2: Intake of selected food groups based on 3-day weighed food records in women and men of raw food eaters, RBVD vegans and RBVD omnivores.
