# Supplementary material for: Nutritional Intake and Biomarker Status in Strict Raw Food Eaters
Source: Nutrients. 2022 Apr 21;14(9):1725. doi: 10.3390/nu14091725 (PMC9105765; doi:10.3390/nu14091725)
Supplement: Supplementary file 1 [file nutrients-14-01725-s001.zip › nutrients-1673246-supplementary.pdf]

Supplement:

Nutritional intake and biomarker status in strict raw food eaters

Klaus Abraham [1*], Iris Trefflich [1], Fabian Gauch [1], and Cornelia Weikert [1]

German Federal Institute for Risk Assessment, Department of Food Safety, 10589 Berlin, Germany

Supplemental Table S1. Nutrient intake based on 3-day weighed food records in women and men of raw food eaters, RBVD vegans and RBVD omnivores

	Women			Men			Reference Values D-A-CH*
	Raw food (n=5)	Vegan (n=18)	Omnivore (n=14)	Raw food (n=10)	Vegan (n=14)	Omnivore (n=13)	
Energy (J/d)	5.6 (4.0-6.5)[a,b,c]	8.1 (7.1-9.9)[a,b]	9.1 (8.1-10.0)[a,c]	9.6 (8.1-13.0)	10.7 (9.6-13.2)	10.2 (9.1-12.1)	
Fiber (g/d)	39 (35-43)[a]	42 (34-55)[a,d]	23 (18-31)[a,d]	62 (53-79)[a,c]	52 (43-62)[a,d]	26 (23-30)[a,c,d]	≥30 mg/d
Protein (g/d)	38 (27-65)[a,c]	61 (49-86)[a]	79 (69-90)[a,c]	79 (38-109)	94 (71-128)	106 (72-127)	
Carbohydrate (g/d)	119 (93-153)[a,b]	231 (208-270)[a,b]	205 (186-229)[a]	210 (186-297)[a]	351 (270-400)[a,d]	231 (212-282)[a,d]	
Saccharose (g/d)	35 (30-49)	48 (36-58)	45 (38-54)	77 (53-105)[a,c]	59 (39-79)[a]	35 (29-56)[a,c]	
Fat (g/d)	31 (22-74)[a,c]	80 (62-109)[a]	98 (89-112)[a,c]	116 (76-154)	95 (83-107)	129 (90-155)	30% Energy
Retinol equ. (µg/d)	1538 (958-3432)	2126 (1336-2674)	1455 (1052-1728)	3849 (1656-5052)[a,b,c]	1204 (806-2002)[a,b]	1340 (1101-2091)[a,c,]	F: 700; M: 850
Vitamin B12 (µg/d)	0.01 (0.00-0.10)[a,c]	0.21 (0.10-0.34)[a,d]	4.83 (4.03-5.77)[a,c,d]	1.78 (0.07-7.96)[a]	0.48 (0.16-0.95)[a,d]	7.72 (5.08-9.96)[a,d]	4.0
Vitamin D (µg/d)	0.65 (0.29-2.99)	0.97 (0.34-1.42)	2.08 (1.07-2.51)	6.03 (3.02-12.11)[a,b]	0.87 (0.21-1.95) [a,b,d]	3.05 (2.19-4.61)[a,d]	20
Folic acid (µg/d)	396 (272-412)[a]	440 (309-602)[a,d]	294 (244-341)[a,d]	553 (474-1168)[a,c]	473 (354-682)[a]	326 (260-370)[a,c]	300
Vitamin B1 (mg/d)	1.08 (0.82-1.18)	1.74 (1.10-2.01)	1.13 (0.98-1.45)	1.89 (1.28-3.05)	1.77 (1.51-2.27)	1.54 (1.22-1.90)	F: 1.0; M: 1.2-1.3
Vitamin B2 (mg/d)	1.19 (0.97-1.33)	1.27 (0.94-1.93)	1.87 (1.55-2.01)	1.89 (1.64-3.08)	1.84 (1.19-2.21)	2.15 (1.87-2.40)	F: 1.0-1.1; M: 1.3-1.4
Niacin equ. (mg/d)	10.5 (5.4-16.3)[a,c]	13.2 (10.9-18.4)[a]	17.5 (15.1-22.3)[a,c]	23.3 (15.7-29.2)	14.6 (13.1-20.8)	18.9 (15.0-34.3)	F: 11-13; M: 15-16
Vitamin B6 (mg/d)	2.05 (1.08-2.25)	2.07 (1.64-2.69)	1.63 (1.29-1.77)	3.33 (2.85-5.43)[a,b,c]	2.37 (2.14-2.99)[a,b]	2.04 (1.55-2.44)[a,c]	F: 1.4; M: 1.6
Vitamin C (mg/d)	315 (220-320)	173 (120-276)	131 (98-217)	401 (273-481)[a,b,c]	218 (108-317)[a,b]	145 (120-176)[a,c]	F: 95; M: 110
Vitamin E (mg/d)	17.5 (9.0-24.2)[a]	24.1 (15.0-37.2)[a,d]	12.4 (10.9-17.1)[a,d]	27.7 (16.5-40.9)[a]	26.6 (17.5-41.0)[a,d]	13.9 (11.4-18.8)[a,d]	F: 12; M: 13-15
Vitamin K (µg/d)	167 (72-227)[a]	265 (171-390)[a,d]	96 (67-338)[a,d]	250 (188-336)[a,c]	250 (149-473)[a,d]	129 (77-175)[a,c,d]	F: 60-65; M: 70-80
Panthothenic acid (mg/d)	3.99 (3.65-6.37)	4.35 (3.64-5.07)	4.92 (4.17-7.17)	9.83 (5.92-15.83)[a,b,c]	4.51 (4.15-6.30)[a,b]	5.38 (4.48-7.58)[a,c]	5
Potassium (g/d)	3.56 (3.41-4.33)	4.06 (3.17-4.94)	3.17 (2.87-3.53)	6.69 (5.20-8.44)[a,b,c]	4.79 (3.64-6.22)[a,b]	3.36 (3.08-4.26)[a,c]	4
Calcium (mg/d)	561 (365-632)[a,c]	852 (652-1127)[a]	973 (803-1259)[a,c]	710 (653-1032)	1156 (707-1439)	1179 (835-1461)	1000
Magnesium (g/d)	407 (289-512)[a]	619 (470-661)[a,d]	367 (306-443)[a,d]	742 (466-931)[a]	714 (606-904)[a,d]	486 (373-536)[a,d]	F: 300; M: 350
Zinc (mg/d)	4.5 (2.8-6.8)[a,b,c]	10.0 (7.4-12.5)[a,b]	11.6 (9.8-12.8)[a,c]	13.1 (7.5-18.7)	11.8 (10.7-14.5)	15.3 (12.2-19.3)	F: 7-10; M: 11-16
Iodine (µg/d)	49.6 (37.4-64.6)[a,c]	74.8 (48.9-124.6)[a]	100.5 (76.5-142.6)[a,c]	78.9 (51.7-106.0)[a]	88.0 (41.6-104.5)[a,d]	165.9 (106.2-181.5)[a,d]	F: 150; M:180-200
Iron (mg/d)	10.0 (9.5-15.1)[a]	20.7 (15.4-23.9)[a,d]	13.2 (11.6-17.3)[a,d]	22.4 (15.8-29.7)[a]	25.0 (19.0-30.2)[a,d]	14.8 (13.7-21.5)[a,d]	F: 10-15; M:10

Data presented as median (Q1 –Q3). Values sharing a common superscript letter were significantly different from each other. ANOVA with post hoc analysis ($p < 0.05$): a) significant differences within sex strata; b)-d): significant differences between type of diet.

* D-A-CH reference values: common reference values of the nutrition societies of the three D-A-CH countries (Germany [D], Austria [A], and Switzerland [CH]). F: women; M: men [17]

Supplement Table S2. Intake of selected food groups based on 3-day weighed food records in women and men of raw food eaters, RBVD vegans and RBVD omnivores

Food group	Women			Men		
	**Raw food (n=5) (g/d)**	**Vegan (n=18) (g/d)**	**Omnivore (n=14) (g/d)**	**Raw food (n=10) (g/d)**	**Vegan (n=14) (g/d)**	**Omnivore (n=13) (g/d)**
Raw vegetables	310 (292-482)	217 (94-426)	108 (60-145)	566 (227-671)[a,b,c]	119 (75-255)[a,b]	81 (41-193)[a,c]
Cabbage family	0.0 (0.0-51.7)	9.8 (0.0 -36.7)	0.0 (0.0-23.0)	8.8 (0.0-26.0)	48.2 (0.0-83.7)	0.0 (0.0-56.3)
Mushrooms	8.7 (0.0-31.0)	0.0 (0.0-21.3)	0.0 (0.0-0.0)	0.0 (0.0-0.0)	0.0 (0.0-6.7)	0.0 (0.0-12.3)
Germinated cereals/legumes*	0.0 (0.0-4.0)	.	.	0.0 (0.0-173)	.	.
Seaweed / Algae*	0.7 (0.0-1.7)	.	.	0.0 (0.0-1.0)	.	.
Fresh fruit	409 (365-425)[a,c]	202 (103-315)[a]	133 (41-215)[a,c]	906 (587-1268)[a,b,c]	154 (69-191)[a,b]	50 (0-211)[a,c]
Dried fruits	10.0 (0.0-21.0)	1.2 (0.0-8.0)	0.0 (0.0-0.0)	38.0 (0.0-149.7)[a,c]	5.8 (0.0-28.7)[a,d]	0.0 (0.0-0.0)[a,c,d]
Fruit juice	37.7 (0.0-40.0)	32.8 (1.3-125.3)	27.8 (0.0-115.3)	19.2 (0.0-79.7)	11.2 (0.0-103.7)	76.7 (0.0-162.7)
Nuts/Seeds	32.3 (13.3-56.7)	15.3 (4.3-39.0)	4.7 (0.0-23.7)	64.8 (0.0- 218.3)	20.3 (1.3-37.3)	0.7 (0.0-19.0)
Fat (plant based)	9.0 (0.7-20.7)	16.2 (11.7-26.0)	11.5 (7.7-14.0)	0.0 (0.0-49.0)	23.2 (8.0-34.0)	10.0 (8.7-17.7)
Fat (animal based)	0.0 (0.0-0.0)[a,c]	0.0 (0.0-0.0)[a,d]	15.0 (5.3-22.7)[a,c,d]	0.0 (0.0-0.0)[a]	0.0 (0.0-0.0)[a,d]	16.0 (0.0-25.0)[a,d]
Meat	0.0 (0.0-0.0)[a,c]	0.0 (0.0-0.0)[a,d]	83.8 (42.0-131.3)[a,c,d]	2.3 (0.0-56.7)[a,b]	0.0 (0.0-0.0)[a,b,d]	72.3 (0.0-132.7)[a,d]
Fish	0.0 (0.0-0.0)	0.0 (0.0-0.0)	0.0 (0.0-0.0)	0.0 (0.0-0.0)[a]	0.0 (0.0-0.0)[a,d]	0.0 (0.0-40.3)[a,d]
Eggs	0.0 (0.0-0.0)[a,c]	0.0 (0.0-0.0)[a,d]	17.2 (6.3-27.3)[a,c,d]	0.0 (0.0-0.0)[a]	0.0 (0.0-0.0)[a,d]	22.7 (8.7-55.3)[a,d]
Low-fat dairy products	0.0 (0.0-0.0)[a,c]	0.0 (0.0-0.0)[a,d]	239.2 (119.0-328.3)[a,c,d]	0.0 (0.0-0.0)[a]	0.0 (0.0-0.0)[a,d]	259.3 (164.3-429.0)[a,d]
Cooked vegetables**	.	178 (134-241)	152 (71-211)	.	178 (81-283)	91 (51-177)
Cooked legumes**	.	51 (4-104)[a]	0 (0-14)[a]	.	51 (0-88)[a]	0 (0-8)[a]
Bread**	.	73 (36-111)	88 (72-119)	.	107 (56-149)	140 (70-175)
Pasta, rice, grain**	.	70 (23-162)	48 (0-117)	.	114 (73-268)	83 (0-130)

Data presented as median with interquartile range (Q1-Q3). Values sharing a common superscript letter were significantly different from each other. ANOVA with post hoc analysis ($p < 0.05$): a) significant differences within sex strata; b)-d): significant differences between type of diet.
Food groups marked with * were not assessed in the RBVD study or with ** were not consumed by the raw food eaters.
